# Genetic variability in the expression of the SARS-CoV-2 host cell entry factors across populations

**DOI:** 10.1038/s41435-020-0107-7

**Published:** 2020-08-06

**Authors:** Lourdes Ortiz-Fernández, Amr H Sawalha

**Affiliations:** 1 Department of Pediatrics, University of Pittsburgh, Pittsburgh, Pennsylvania, USA; 2 Department of Medicine, University of Pittsburgh, Pittsburgh, Pennsylvania, USA

**Keywords:** SARS-CoV-2, COVID-19, Genetic, ACE2, TMPRSS2, eQTL

## Abstract

The entry of SARS-CoV-2 into host cells is dependent upon angiotensin-converting enzyme 2 (ACE2), which serves as a functional attachment receptor for the viral spike glycoprotein, and the serine protease TMPRSS2 which allows fusion of the viral and host cell membranes. We devised a quantitative measure to estimate genetic determinants of *ACE2* and *TMPRSS2* expression and applied this measure to >2,500 individuals. Our data show significant variability in genetic determinants of *ACE2* and *TMPRSS2* expression among individuals and between populations, and indicate a genetic predisposition for lower expression levels of both key viral entry genes in African populations. These data suggest that host genetics related to viral entry mechanisms might influence inter-individual variability in disease susceptibility and severity of COVID-19.

## Introduction

The severe acute respiratory syndrome coronavirus 2 (SARS-CoV-2) is a novel single-stranded RNA virus of the *Coronaviridae* family. This recently emerged virus is the cause of a pandemic infection that can result in severe life-threatening disease (Coronavirus Disease-2019; COVID-19)^1, 2^. Similar to SARS-CoV (which caused SARS), SARS-CoV-2 entry into target host cells is mediated through binding of the viral spike glycoprotein to angiotensin-converting enzyme 2 (ACE2) on the cell surface^3–6^. The interaction between coronaviral spike proteins and their attachment receptor ACE2 is believed to be key for viral transmissibility and dissemination of infection to organs and tissues^7^. In addition, the host cell serine protease TMPRSS2 cleaves the spike protein of SARS-CoV-2 to allow fusion of the viral and host cell membranes, which is an essential step in viral entry^3, 8, 9^. Other molecules have been proposed to play alternative roles in the SARS-CoV-2 entry mechanisms. It has been suggested that cathepsin B and L may cleave the spike protein in the absence of TMPRSS2^9^. The results of other studies proposed that SARS-CoV-2 entry is primarily mediated through endocytosis and that PIKfyve, TPC2, and cathepsin L but not cathepsin B are essential for viral entry^5, 8^. In addition, it has been described that furin may have an effect along with TMPRSS2 and cathepsins on activating viral entry^8^. However, a previous study revealed inconclusive results since furin pre-activation enhanced or reduced SARS-CoV-2 pseudovirus entry in different cell types^6, 8^. These data suggest that SARS-CoV-2 may use alternative mechanisms to enter the cells but the role of these mechanisms and molecules involved is less clear and needs further investigation.

To gain insight into genetic determinants of SARS-CoV-2 transmissibility and potential viremia and disseminated infection, we devised a quantitative measure to assess the cumulative effect of genetic variants upon the expression of the two key molecules involved in SARS-CoV-2 viral entry, *ACE2* and *TMPRSS2*, and then applied this measure to 2,504 individuals from 5 different populations around the world.

## Results and Discussion

**Figure 1:**
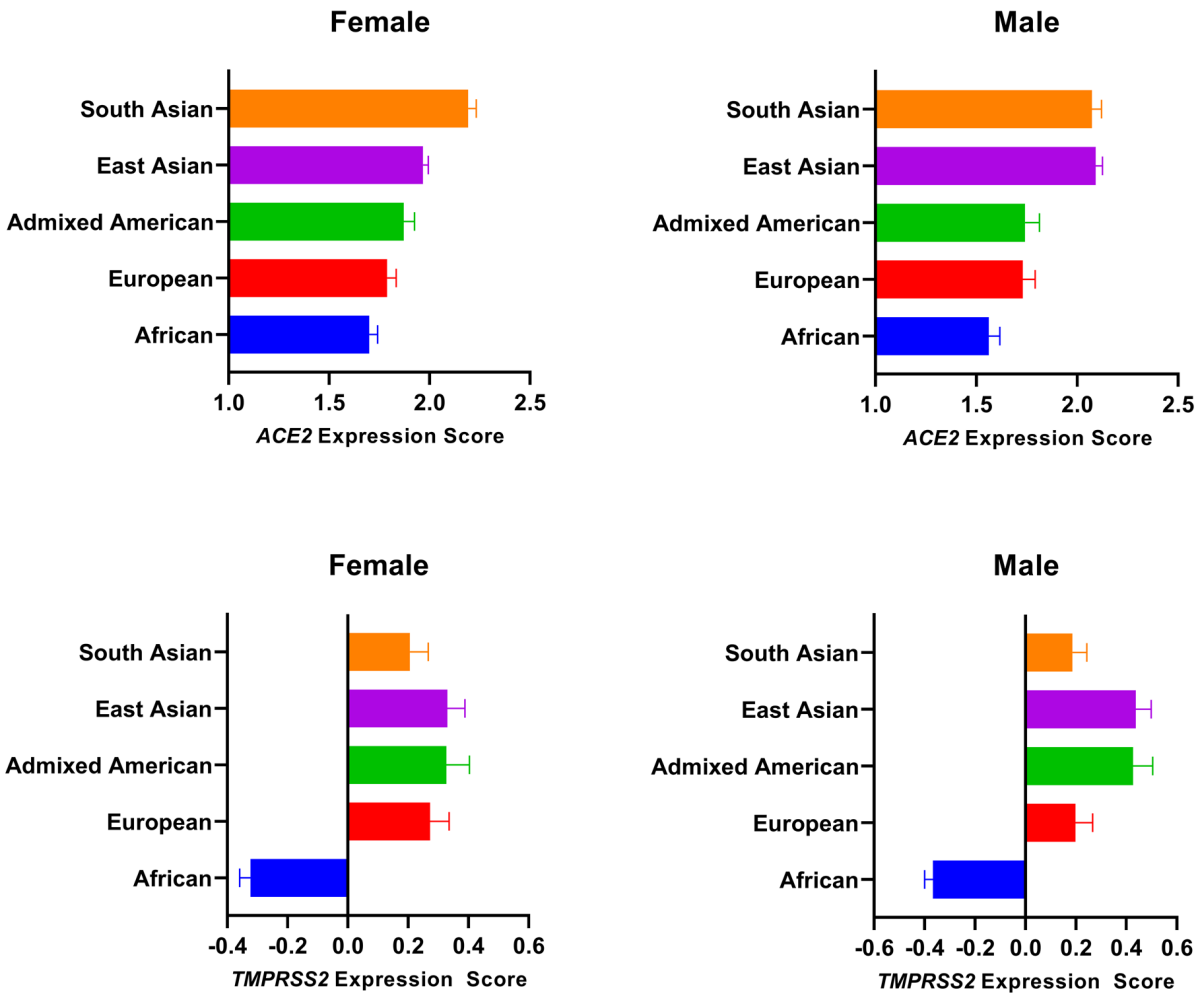
Differences in the cumulative effect of genetic polymorphisms on the expression of SARS-CoV-2 entry mediators *ACE2* and *TMPRSS2* between populations. upper left Genetic expression score of *ACE2* in female individuals. ANOVA *P* <0.0001. Adjusted *P* values using Tukey’s multiple comparisons test: African vs. European, *P*= 0.47; African vs. Admixed American, *P*= 0.043; African vs. East Asian, *P* <0.0001; African vs. South Asian, *P* <0.0001; European vs. Admixed American, *P*= 0.71; European vs. East Asian, *P*= 0.0175; European vs. South Asian, *P* <0.0001; Admixed American vs. East Asian, *P*= 0.56; Admixed American vs. South Asian, *P* <0.0001; East Asian vs. South Asian, *P*= 0.0017. upper right Genetic expression score of *ACE2* in male individuals. ANOVA *P* <0.0001. Adjusted *P* values using Tukey’s multiple comparisons test: African vs. European, *P*= 0.14; African vs. Admixed American, *P*= 0.17; African vs. East Asian, *P* <0.0001; African vs. South Asian, *P* <0.0001; European vs. Admixed American, *P*= 1; European vs. East Asian, *P* <0.0001; European vs. South Asian, *P* <0.0001; Admixed American vs. East Asian, *P*= 0.0004; Admixed American vs. South Asian, *P*= 0.0009; East Asian vs. South Asian, *P*= 1. lower left Genetic expression score of *TMPRSS2* in female individuals. ANOVA *P* <0.0001. Adjusted *P* values using Tukey’s multiple comparisons test: African vs. European, *P* <0.0001; African vs. Admixed American, *P*< 0.0001; African vs. East Asian, *P*< 0.0001; African vs. South Asian, *P* <0.0001; European vs. Admixed American, *P*= 0.97; European vs. East Asian, *P*= 0.95; European vs. South Asian, *P=* 0.93; Admixed American vs. East Asian, *P*= 1; Admixed American vs. South Asian, *P*= 0.66; East Asian vs. South Asian, *P*= 0.55. lower right Genetic expression score of *TMPRSS2* in male individuals. ANOVA *P* <0.0001. Adjusted *P* values using Tukey’s multiple comparisons test: African vs. European, *P* <0.0001; African vs. Admixed American, *P* <0.0001; African vs. East Asian, *P* <0.0001; African vs. South Asian, *P* <0.0001; European vs. Admixed American, *P*= 0.084; European vs. East Asian, *P*= 0.03; European vs. South Asian, *P=* 1; Admixed American vs. East Asian, *P*= 1; Admixed American vs. South Asian, *P*= 0.053; East Asian vs. South Asian, *P*= 0.015.

We calculated a cumulative genetic expression score (GES) for *ACE2* and *TMPRSS2,* as a measure to estimate host genetic determinants of viral entry of SARS-CoV-2. We evaluated this measure in 2,504 individuals across the 5 major populations included in the 1000 Genomes project (African, Admixed American, European, East Asian, and South Asian)^10^. Because *ACE2* is located on the X chromosome, we analyzed female and male individuals separately. There was a significant difference in the cumulative genetic expression score of *ACE2* between populations (ANOVA *P* <0.0001 for both male and female groups). Genetic determinants of highest expression of *ACE2* were observed in South Asian and East Asian populations, while African populations were genetically associated with the lowest *ACE2* expression levels (Figure 1).

Similarly, significant differences for *TMPRSS2* were observed in both female and male groups across populations (ANOVA *P* <0.0001). East Asian populations had the highest values for genetic determinants of *TMPRSS2* expression, and Africans showed genetic predisposition for the lowest *TMPRSS2* expression levels across populations (Figure 1).

As mentioned earlier, *ACE2* is located on the X chromosome (and not on the pseudoautosomal region). Therefore, female individuals will have two copies of the gene while males will only have one. Normally, X-chromosome genes are subject to random X-chromosome inactivation, silencing one gene copy in females to keep gene expression balance between females and males. However, a number of X-chromosome genes, including *ACE2*, are known to escape X-chromosome inactivation^11^. We did not observe differences in genetically determined *ACE2* expression between male and female individuals. Previous reports showed higher expression of *ACE2* in male compared to female tissues, and that this difference in expression was predominantly attributed to non-genetic factors, consistent with our findings^11, 12^. No difference between male and female individuals for *TMPRSS2* were observed in our study.

These data suggest that genetic determinants of *ACE2* and *TMPRSS2* expression might play a role in the variability of transmission and severity of SARS-CoV-2 between populations. African populations showed a genetic predisposition for lower expression levels of both *ACE2* and *TMPRSS2*, which are vital for SARS-CoV-2 entry into host cells. These data suggest that a genetic component might contribute to lower numbers of reported COVID-19 in Africa. However, it remains likely that non-genetic factors such as age and associated comorbidities might play a more important role than host genetic elements, especially in determining disease severity and outcome in infected individuals. In addition, genome-wide association studies will be needed to characterize genetic susceptibility to a more severe disease course in patients infected with COVID-19. Additional studies to replicate and extend our findings and examine expression levels of ACE2 and TMPRSS2 in different cell types across populations and in patients infected with COVID-19 are warranted.

## Methods

We devised a cumulative genetic expression score to estimate genetically determined expression of *ACE2* and *TMPRSS2*. We used expression quantitative trait loci (eQTL) data for *ACE2* and *TMPRSS2* in tissues included in the Genotype-Tissue Expression project (GTEx, release V8)^13^. All eQTL variants affecting *ACE2* and *TMPRSS2* expression across all cell types and tissues were identified, and then pruned to remove variants in linkage disequilibrium (LD). LD pruning was performed using PLINK v.1.9 and the combined haplotypes of the 1000 Genomes Project populations^14^. For variants that demonstrate eQTL effects in multiple tissues, the most significant eQTL normalized effect size value was used. The genetic variants used to calculate GRES values for *ACE2* and *TMPRSS2* are shown in Supplementary Dataset 1. A total of 21 genetic polymorphisms that affect *ACE2* expression, and 14 that affect *TMPRSS2* expression were identified and used in subsequent analysis. The cumulative genetic expression score was derived using the formula: $\text{GES=}\sum_{\text{i=1}}^{x}\left({\text{n}}_{\text{i}}\text{×}{\text{NES}}_{\text{i}}\right)$, where n is the number of alternative alleles (0, 1 or 2), x is the number of evaluated variants in *ACE2* and *TMPRSS2*, and NES is the normalized effect size which reflects the expression in the alternative allele relative to the reference allele for each variant. We calculated the genetic expression scores for *ACE2* and *TMPRSS2* in a total of 2,504 individuals from the five major populations included in the 1000 Genomes Project phase 3 release: African, n=661; Admixed American, n=347; East Asian, n=504; European, n=503; and South Asian, n=489^10^. To determine if the cumulative genetic expression score was different across populations, one-way ANOVA following by Tukey’s multiple comparison test was performed using GraphPad Prism version 8.1.1 (GraphPad Software, La Jolla California USA). ANOVA p values < 0.05 and Tukey’s adjusted p values < 0.05 were considered significant.

## Supplementary Material

1

corrected text version
